# Engineering Tumour Cell-Binding Synthetic Polymers with Sensing Dense Transporters Associated with Aberrant Glutamine Metabolism

**DOI:** 10.1038/s41598-017-06438-y

**Published:** 2017-07-20

**Authors:** Naoki Yamada, Yuto Honda, Hiroyasu Takemoto, Takahiro Nomoto, Makoto Matsui, Keishiro Tomoda, Masamitsu Konno, Hideshi Ishii, Masaki Mori, Nobuhiro Nishiyama

**Affiliations:** 1 Laboratory for Chemistry and Life Science, Institute of Innovative Research, Tokyo Institute of Technology, 4259 Nagatsuta, Midori-ku, Yokohama, Kanagawa 226-8503 Japan; 2 Graduate School of Medicine, Department of Gastroenterological Surgery, Osaka University, 2-2 Yamadaoka, Suita, Osaka 565-0871 Japan; 3 Innovation Center of Nanomedicine (iCONM), Kawasaki Institute of Industrial Promotion, 3-25-14 Tonomachi, Kawasaki-ku, Kawasaki, Kanagawa 210-0821 Japan

## Abstract

Increased glutamine uptake toward the elevated glutaminolysis is one of the hallmarks of tumour cells. This aberrant glutamine metabolism has recently attracted considerable attention as a diagnostic and therapeutic target. Herein, we developed glutamine-functionalized polymer to achieve a selective high affinity to tumour cells overexpressing glutaminolysis-related transporter ASCT2. In *in vitro* study, our developed polymer exhibited faster and higher cellular uptake in tumour cells than that in normal cells. Uptake inhibition study revealed the dominant contribution of ASCT2 to the polymer-cell interaction. Furthermore, the binding affinity of the polymer to tumour cells was estimated to be comparable to that of the potent ligand molecules reported in the literature. In *in vivo* study, the polymer showed prolonged retention at tumour site after intratumoral injection. This study offers a novel approach for designing tumour cell-binding synthetic polymers through the recognition of dense transporters related to tumour-associated metabolism.

## Introduction

Tumour cells exhibit distinctive metabolic activities compared to normal differentiated cells because of their genetic and epigenetic alteration^1, 2^ One of the major metabolic pathways in tumour cells is a high rate of glycolysis even in the presence of oxygen, also known as Warburg effect^1, 3^. Although the Warburg effect was first described in 1924^2, 3^, other tumour-related metabolic alterations such as lipid synthesis, fatty acid oxidation, and glutamine metabolism, have been revealed during the last decade. In addition, recent advances in metabolomics, which is the comprehensive analysis of the metabolite, have provided in-depth understanding of these metabolic activities. Owing to these recent efforts, tumour-related metabolisms have been recently recognized as one of the hallmarks of tumour cells, and thus have been attracted much attention as a therapeutic and diagnostic target.

Among tumour-related metabolisms, elevated glutaminolysis plays a critical role for tumour growth and survival by supporting macromolecular biosynthesis, ATP production, and redox balance regulation^4, 5^. To satisfy the increased demand of glutamine from elevated glutaminolysis, tumour cells overexpress glutamine transporters. In particular, system ASC transporter 2 (ASCT2) has been demonstrated to be overexpressed on various tumour cells including hepatocellular carcinoma^6^, prostate cancer^7^, and breast cancer^8^. In addition, inhibition of ASCT2 function has resulted in a decrease of glutamine uptake and suppression of tumour cell growth^7–9^, indicating the dominant contribution of ASCT2 for glutamine uptake in tumour cells and tumour growth.

Focusing on increased glutamine uptake by ASCT2 in tumour cells, glutamine has been utilized as an imaging agent like ^18^F-fluorodeoxyglucose, which has been clinically used as a powerful diagnosis tool to visualize the malignant tissues possessing the augmented glucose uptake. Previous studies have indeed demonstrated the successful *in vivo* tumour imaging using glutamine analogue PET probes^10, 11^. Considering this promising potential, glutamine is expected to be used as an ASCT2-targeting ligand molecule; however, glutamine-based ligand has yet to be developed probably due to weak binding affinity of glutamine to ASCT2. Dissociation constant (*K*_d_) of glutamine to ASCT2 was estimated to be 20 μM^12^, whereas *K*_d_ value of potent ligands to their receptors should be tens of nanomolar range^13^. Thus, chemical design of glutamine-functionalized molecules toward improvement of this weak binding affinity is prerequisite to utilize glutamine as a ligand. In this regard, multivalent effect offers a promising strategy because multiple ligand-transporter interaction can dramatically enhance the binding affinity associated with high transporter density on target cells^14–16^. In addition, such multivalent interaction is unlikely to occur on cell surface with low target receptor density^16^. Thus, it is hypothesized that multivalent glutamine ligand permits selective affinity to tumour cells overexpressing ASCT2.

**Figure 1:**
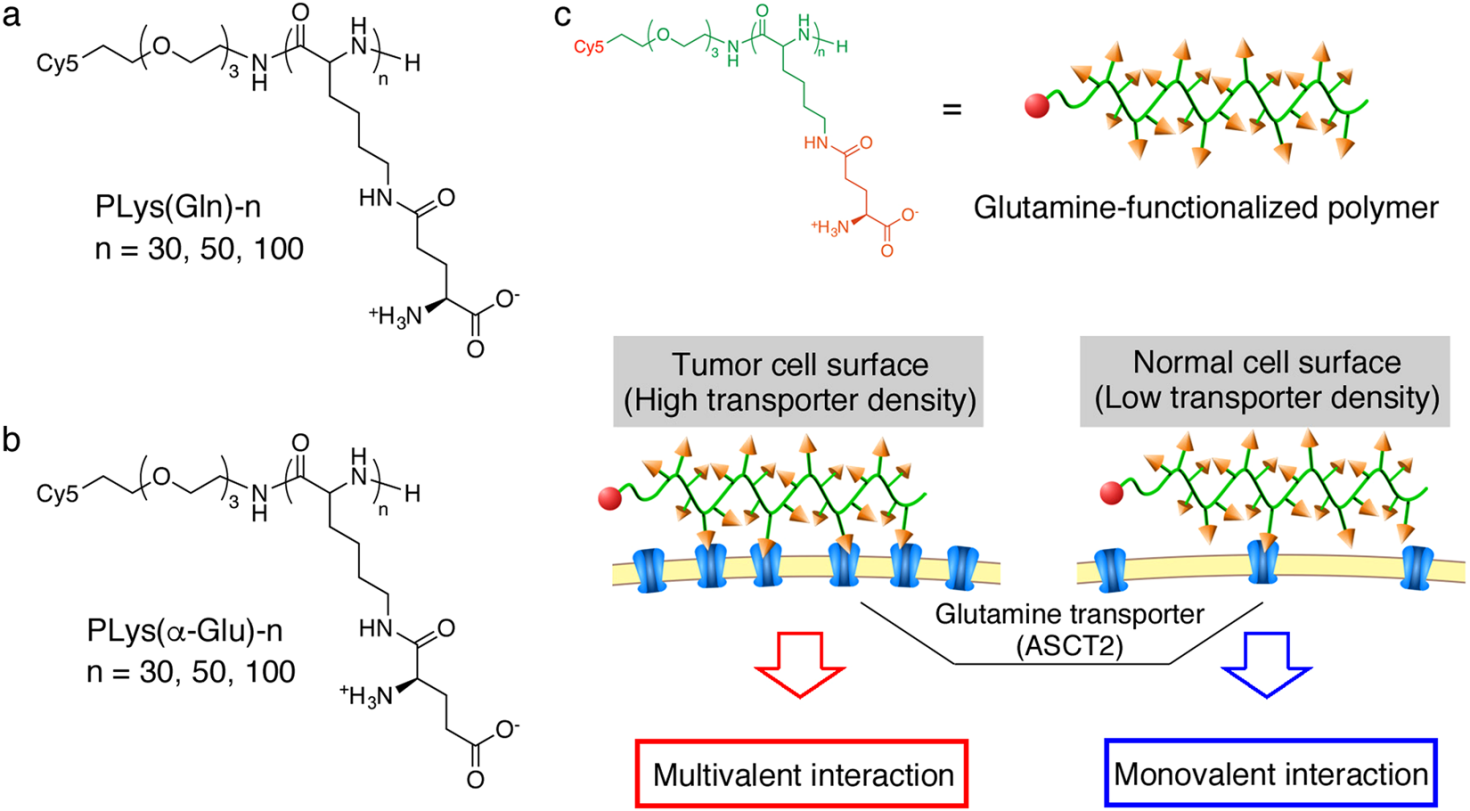
Design of glutamine-functionalized polymer and interaction of the polymer with cell surface. (**a**,**b**) Chemical structure of PLys(Gln)-n (**a**) and PLys(α-Glu)-n (**b**). (**c**) Illustration of interaction between the glutamine-functionalized polymer and cell surface. The polymer strongly interacts with tumour cell surface by multivalent interaction associated with high transporter density. In contrast, the polymer weakly interacts with normal cell surface because of low transporter density.

Herein, on the basis of our hypothesis, we designed poly(L-lysine) derivatives having glutamine moieties at the side chain, and examined whether these glutamine-functionalized polymers can provide a selective high affinity to tumour cells overexpressing ASCT2 as shown in Fig. 1. Our results show that glutamine-functionalized polymer strongly interacted with dense ASCT2 on tumour cells. Moreover, this strong interaction was observed even in *in vivo* tumour tissue.

## Results

### Design and synthesis of glutamine-functionalized polymers

A series of glutamine-functionalized polymers were synthesized by ring-opening polymerization of *N*-carboxyanhydride and subsequent modification of the side chain (Supplementary Fig. S1–S4). Briefly, azide-functionalized poly(L-lysine) was first synthesized by ring-opening polymerization of *N*-carboxyanhydride of ε-trifluoroacetyl-L-lysine and deprotection of trifluoroacetyl group. Then, the side chain was modified to have glutamine moieties through γ-amide linkage. It should be noted that modification of γ-amide group of glutamine is expected to tolerate the transporter-glutamine interaction as reported in a previous study, where glutamine analogues with γ-amide modification strongly inhibited ASCT2 function^17^. Finally, dibenzocyclooctyne modified Cy5 fluorescence dye was reacted with azide terminus of the polymer by copper-free click chemistry, for the detection of polymers by fluorescence in subsequent experiments. The resulting polymers were termed as PLys(Gln)-n (Fig. 1a), where n is the mean degree of polymerization (DP). In this design, polymer length is a crucial factor for multivalent interaction with surface transporters. Accordingly, the polymers having the different length (DP was 30, 50, and 100) were synthesized. Meanwhile, control polymers (termed as PLys(α-Glu)-n (Fig. 1b), n is DP) were prepared in the same manner with minor modification: glutamine moieties were changed to α-glutamyl moieties to investigate the effect of chemical structure of the side chain on binding affinity. Quantitative modification of lysine residue and narrow molecular weight distribution were confirmed for all polymers by ^1^H NMR and size exclusion chromatography, respectively (Supplementary Fig. S5–S12).

### ASCT2 Expression

**Figure 2:**
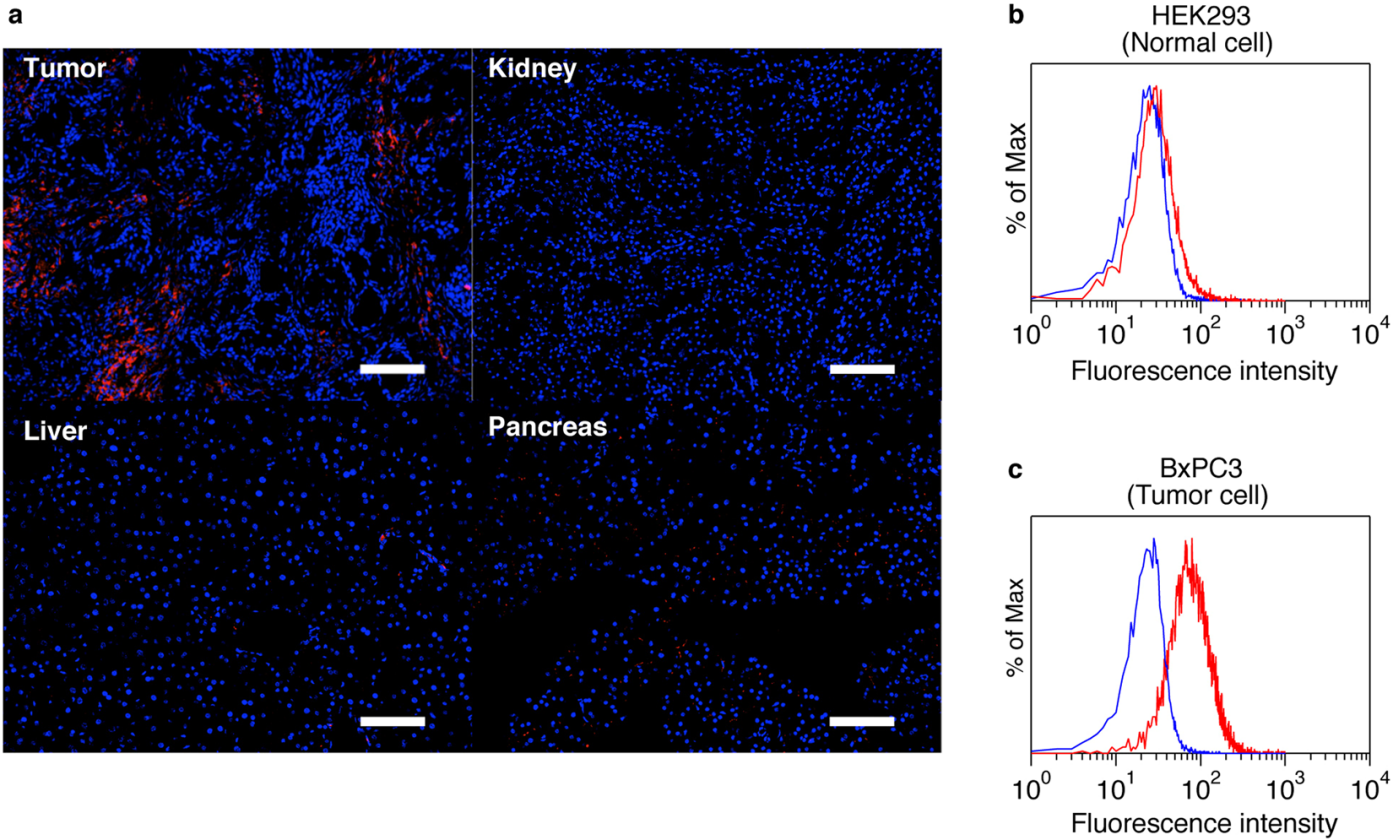
*In vivo* and *in vitro* expression of ASCT2. (**a**) Immunohistochemical analysis of tissues in mice bearing subcutaneous BxPC3 tumours. Red, anti-human/murine ASCT2 antibody; blue, nucleus. Scale bar, 100 μm. (**b**,**c**) Flow cytometric analysis of ASCT2 expression on HEK293 cells (**b**) and BxPC3 cells (**c**). Red, anti-human ASCT2 antibody; blue, isotype control IgG.

We next evaluated the expression of ASCT2 *in vivo* and *in vitro*. Immunohistochemical analysis of a subcutaneous BxPC3 (human pancreatic cancer cell) tumour in a mouse with anti-human/murine ASCT2 antibody revealed that ASCT2 was distinctly overexpressed in the tumour compared to normal tissues (Fig. 2a, Supplementary Fig. S13), suggesting that ASCT2 can be a potential target transporter on cancer cells. Flow cytometric analysis also demonstrated the overexpression of ASCT2 on BxPC3 cells compared to the HEK293 (human embryonic kidney) cells (Fig. 2b,c). In addition, enzyme-linked immunosorbent assay (ELISA) revealed 4.8-fold higher expression of ASCT2 on BxPC3 cells compared to normal HEK293 cells (Supplementary Fig. S14). This overexpression ratio is in line with previous studies, where tumour cells showed approximately 2- to 10-fold higher expression of ASCT2 compared to their counterparts^7, 18, 19^. Thus, we used these cell lines for the assessment of interaction between the developed polymers and ASCT2 in *in vitro* studies.

### Cellular Uptake Analysis

**Figure 3:**
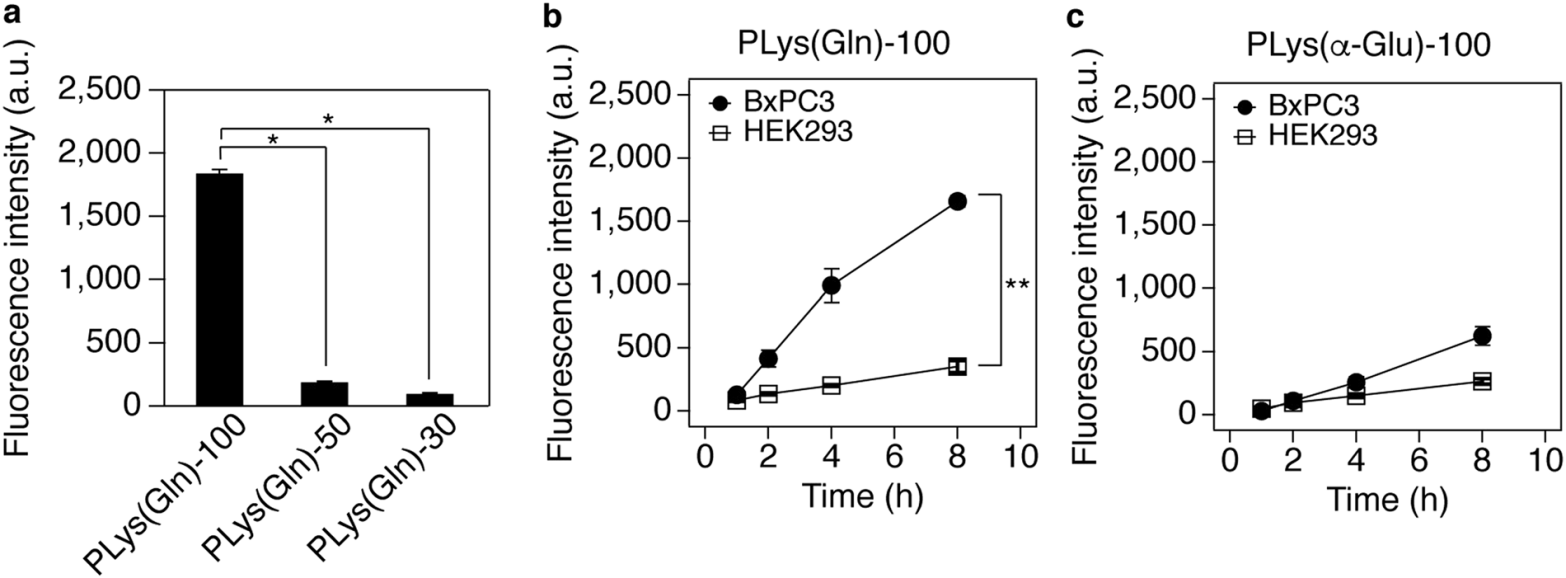
Cellular uptake analysis of the polymers. (**a**) Cellular uptake analysis in BxPC3 cells after 3 h incubation with the polymers. Data are mean ± S.D. (n = 3). p* < 0.001 (one-way ANOVA with Tukey’s multiple comparison test). (**b**,**c**) Time-dependent cellular uptake analysis of PLys(Gln)-100 (**b**) and PLys(α-Glu)-100 (**c**) in BxPC3 and HEK293 cells. Data are mean ± S.D. (n = 3). p** < 0.001 (two-way ANOVA with Tukey’s multiple comparison test).

To examine the cellular interaction of PLys(Gln)-n with cultured tumour cells, the flow cytometric analysis was performed. The cellular uptake was quantified by measuring Cy5 fluorescence intensity from the cells treated with the polymers (Fig. 3a). A series of PLys(Gln)-n exhibited DP-dependent uptake behaviour; PLys(Gln)-100 showed the highest uptake in BxPC3 cells, which was 9.7-fold and 18-fold higher than that of PLys(Gln)-50 and PLys(Gln)-30, respectively. Similar DP-dependent interaction was also observed in HepG2 (human liver cancer) cells (Supplementary Fig. S15), which overexpress ASCT2 (Supplementary Fig. S14, ref. 20). According to a previous study, the interaction potency of multivalent polymeric ligand was exponentially enhanced by an increase of the polymer length^21^. Thus, this drastically high cellular uptake of PLys(Gln)-100 is probably due to the multivalent interaction between the polymer and the tumour cells.

To investigate the tumour specificity of PLys(Gln)-100, the time-dependent cellular uptake was evaluated using HEK293 cells as well as BxPC3 cells (Fig. 3b). PLys(Gln)-100 exhibited faster uptake in BxPC3 cells compared to HEK293 cells. After 8 h incubation, PLys(Gln)-100 uptake in BxPC3 cells was 4.7-fold higher than that in HEK293 cells. These results are consistent with the scheme that PLys(Gln)-100 might strongly interact with tumour cells overexpressing ASCT2 through the strong multivalent effect, while weakly interacting with normal cells because of less binding sites as illustrated in Fig. 1c.

To get insight about the effect of chemical structure on this cellular interaction, we investigated the time-dependent cellular uptake of PLys(α-Glu)-100. This polymer was used as a control polymer because it has same molecular weight and neutral charge with PLys(Gln)-100 with the different position of free amine group at the side chain. As shown in Fig. 3c, PLys(α-Glu)-100 revealed slightly higher uptake in BxPC3 cells compared to HEK293 cells. Considering that glutamate weakly interacted with ASCT2^22, 23^, it could be assumed that PLys(α-Glu)-100 having glutamate-like structure at the side chain might interact with ASCT2 to some extent and exhibit multivalent effect. Consistent with this assumption, a series of PLys(α-Glu)-n also showed DP-dependent uptake behaviour in cultured cells (Supplementary Fig. S16). However, compared to PLys(Gln)-100, PLys(α-Glu)-100 exhibited slower uptake in BxPC3 cells and lower uptake ratio between BxPC3 and HEK293 cells. The relatively high affinity of PLys(Gln)-100 to tumour cells is probably due to its chemical structure at the side chain, which is similar to potent ASCT2 inhibitor having γ-amide modified glutamine structure^17, 24^.

### Transporter Specificity

**Figure 4:**
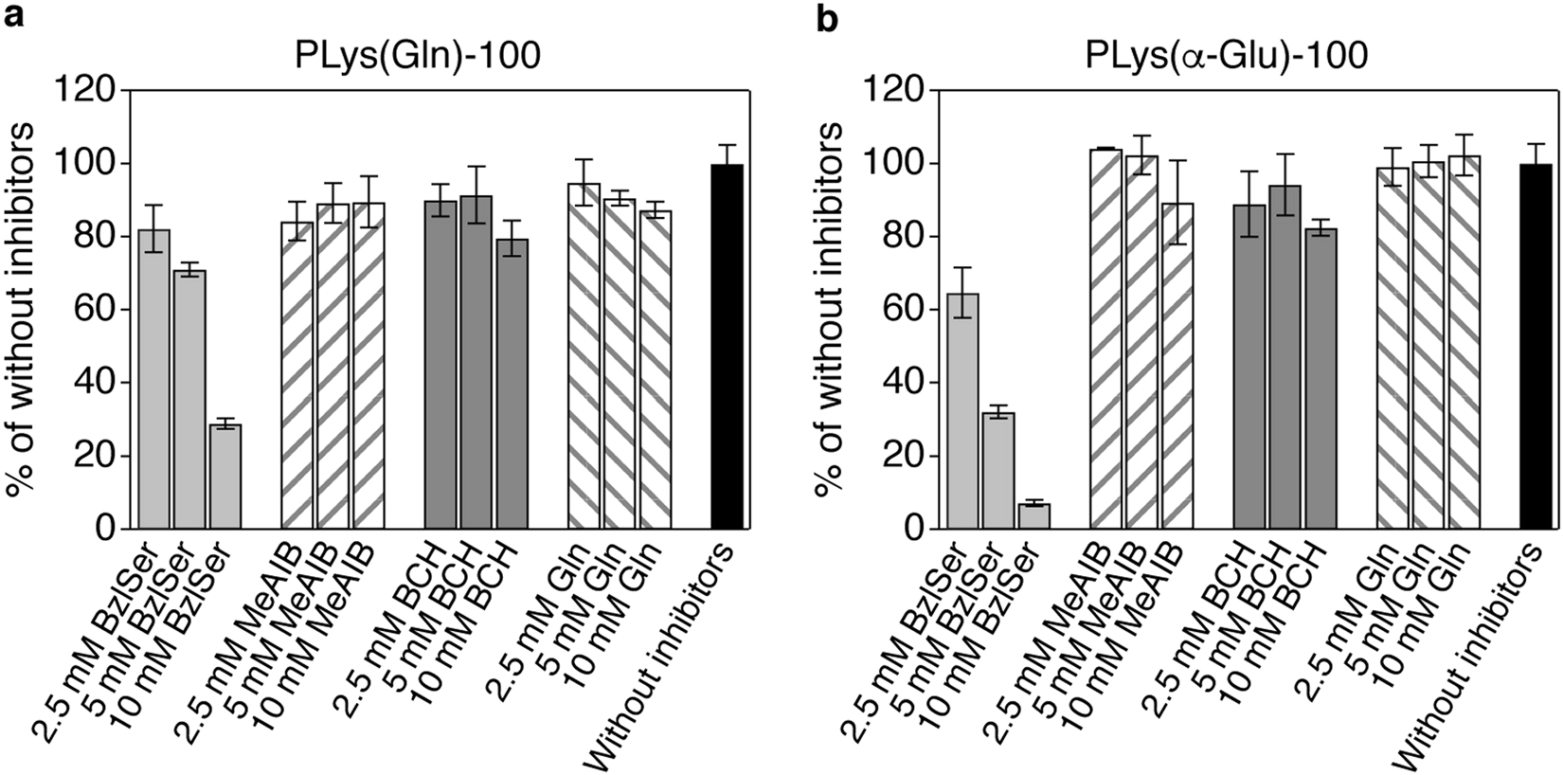
Cellular uptake analysis of PLys(Gln)-100 and PLys(α-Glu)-100 in the presence of transporter inhibitors. (**a**,**b**) Effect of inhibitors on cellular uptake of PLys(Gln)-100 (**a**) and PLys(α-Glu)-100 (**b**) in BxPC3 cells (BzlSer, ASCT2 inhibitor; MeAIB, system A inhibitor; BCH, system L inhibitor; Gln, system N inhibitor). Data are mean ± S.D. (n = 3).

To examine the transporter specificity of the developed polymers, cellular uptake was evaluated with several inhibitors for glutamine transporters: ASCT2, system A, system L, and system N transporters. For their inhibitors, we used *O*-benzyl-L-serine (BzlSer, ASCT2 inhibitor), 2-(methylamino)isobutyric acid (MeAIB, system A inhibitor), 2-amino-2-norbornanecarboxylic acid (BCH, system L inhibitor), and L-glutamine (Gln, system N inhibitor). As shown in Fig. 4a, only BzlSer exhibited strong concentration-dependent inhibitory effect, while the other inhibitors showed negligible inhibitory effect, indicating the dominant contribution of ASCT2 to the uptake of PLys(Gln)-100. Similar inhibitory effects were also observed for PLys(Gln)-50 (Supplementary Fig. S17). Among above-mentioned glutamine transporters, ASCT2 has relatively high affinity with glutamine (Michaelis constant (*K*_m_) is 20–60 μM^12, 23^, whereas the other transporters has *K*_m_ in the range of approximately 150–1600 μM^25–28^). Because of this relatively strong interaction between ASCT2 and glutamine molecule, ASCT2 could be a dominant target transporter for PLys(Gln)-n. Moreover, the overexpression of ASCT2 on BxPC3 cells as shown in Fig. 2c may also contribute to the dominant role of ASCT2 for tumour cell-selective interaction of PLys(Gln)-n. Cellular uptake of PLys(α-Glu)-100 was also inhibited by only BzlSer while its inhibitory effect was higher compared to that of PLys(Gln)-100 (Fig. 4b), suggesting that PLys(α-Glu)-100 modestly interacted with ASCT2.

### Subcellular Distribution of the Polymers

**Figure 5:**
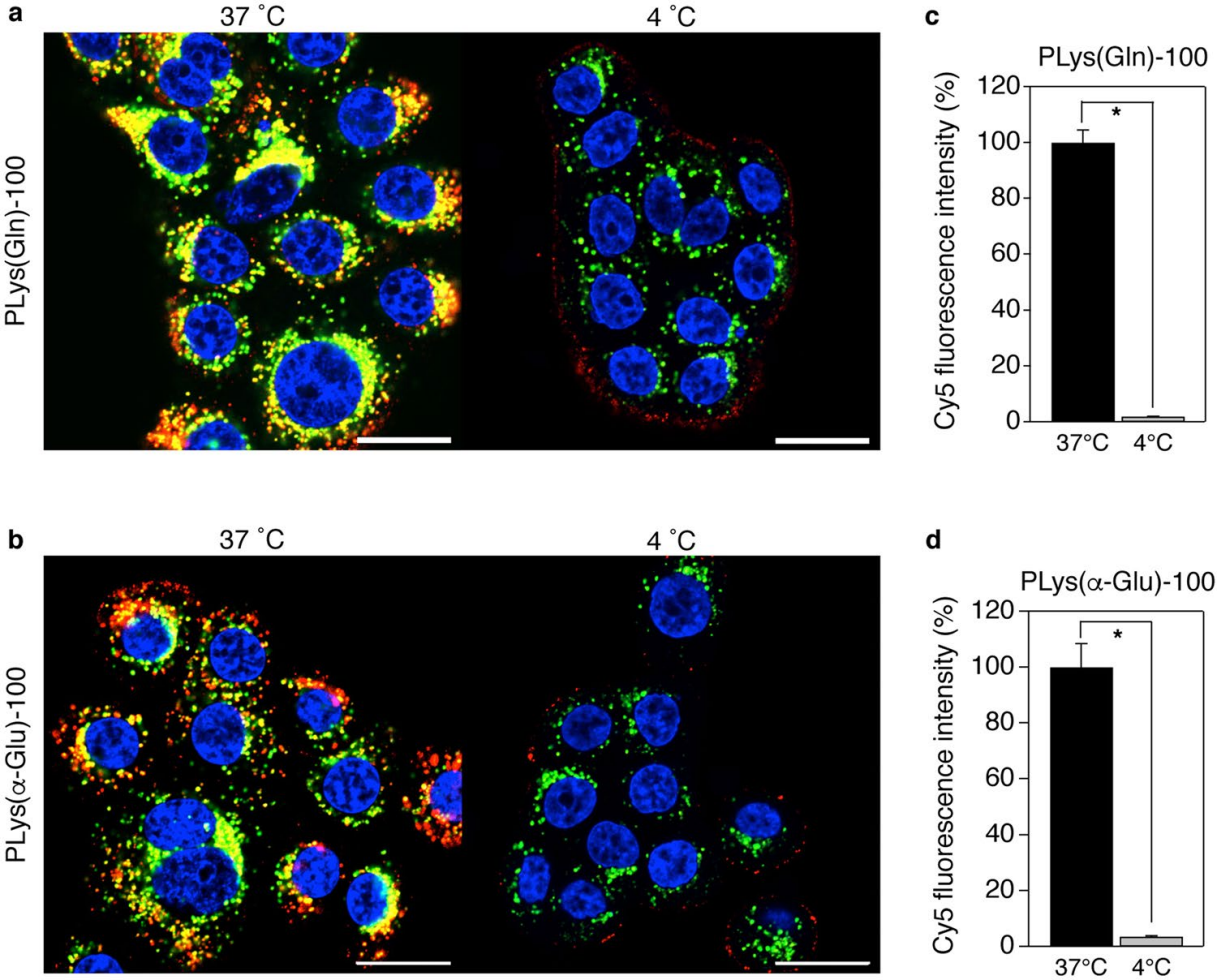
Subcellular distribution of PLys(Gln)-100 and PLys(α-Glu)-100. (**a**,**b**) Confocal laser scanning microscopic images of BxPC3 cells after 3 h treatment with PLys(Gln)-100 (**a**) and PLys(α-Glu)-100 (**b**) at 37 °C (left) and 4 °C (right). Red, Cy5-labeled polymers; green, late endosome/lysosome; blue, nucleus. Scale bar, 20 μm. (**c**,**d**) Cy5 fluorescence intensities of BxPC3 cells after 3 h treatment with PLys(Gln)-100 (**c**) and PLys(α-Glu)-100 (**d**) at 37 °C and 4 °C. The cells were analysed using flow cytometer. Data are mean ± S.D. (n = 3). p* < 0.001 (Student’s *t*-test).

Subcellular distribution of the polymer was then investigated using confocal laser scanning microscopy (CLSM) to assess the internalization of the polymers. As shown in Fig. 5a, in the BxPC3 cells incubated with PLys(Gln)-100 at 37 °C, the polymer (red) was located at intracellular space, and colocalized (yellow) with the late endosome/lysosome indicator, Lysotracker Red (green). In contrast, when BxPC3 cells were incubated with the polymer at 4 °C to suppress the endocytosis, the polymer was located only on the cellular membrane, and did not colocalize with the late endosome/lysosomes. In addition, mean fluorescence intensity from the cells incubated with the polymer at 4 °C was significantly lower compared to that of 37 °C incubation (Fig. 5c). These results indicate that PLys(Gln)-100 first attached to the cell membrane through the polymer-ASCT2 interaction, and the attached polymer was internalized by endocytosis. Note that similar behaviours in subcellular distribution were observed for PLys(α-Glu)-100 and PLys(Gln)-50 (Fig. 5b,d, Supplementary Fig. S18), indicating that these polymers were also internalized by endocytosis after interacting with ASCT2 on cell surface.

### Binding Affinities of the Polymers to ASCT2 on Tumour Cells

To evaluate the binding affinity of the polymers to ASCT2, cell-based competitive inhibition study was carried out using BzlSer. Because BzlSer is a competitive inhibitor for ASCT2 with an inhibition constant (*K*_i_) of 0.9 mM^29^, apparent *K*_d_ values of these polymers can be estimated by assuming a simple inhibition model^30^. PLys(Gln)-100 showed apparent *K*_d_ value of 62 nM, which is comparable to the *K*_d_ value of potent ligands^13^. By contrast, *K*_d_ for PLys(Gln)-50 could not be estimated in this method since the inhibition curve of PLys(Gln)-50 indicated incomplete inhibition within the examined BzlSer concentration (Supplementary Fig. S19). Meanwhile, PLys(α-Glu)-100 showed apparent *K*_d_ value of 250 nM. The *K*_d_ values of PLys(Gln)-100 and PLys(α-Glu)-100 were consistent with cellular uptake behaviour as shown in Fig. 3.

### *In Vivo* Tumour Retention

**Figure 6:**
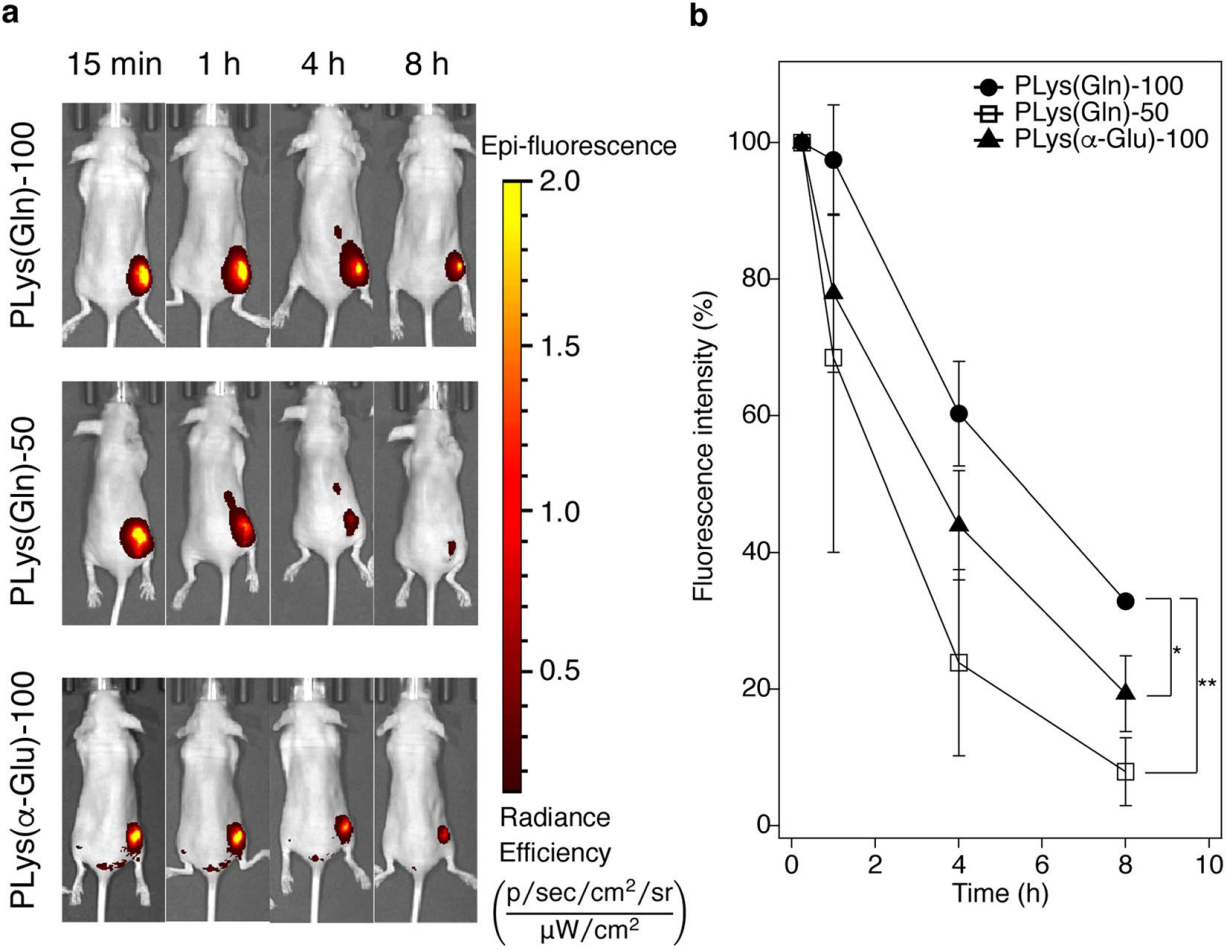
*In vivo* tumour retention of the polymers after intratumoral injection. (**a**) Representative images of mice bearing subcutaneous BxPC3 tumours after intratumoral injection of the polymers. Images were taken by *in vivo* imaging system (IVIS). (**b**) Cy5 fluorescence intensities from the intratumorally injected polymers at subcutaneous BxPC3 tumour. Data are mean ± S.D. (n = 3). p* < 0.05, p** < 0.01 (two-way ANOVA with Tukey’s multiple comparison test).

Finally, to examine *in vivo* binding ability, the polymers were intratumorally injected to subcutaneous BxPC3 tumours in mice, and their retention in the tumour was evaluated by measuring fluorescence intensity at tumour site using *in vivo* imaging system (Fig. 6). PLys(Gln)-50 was most rapidly eliminated from the tumour because PLys(Gln)-50 had low binding affinity to ASCT2 on BxPC3 cells as discussed above. Compared with PLys(α-Glu)-100, PLys(Gln)-100 exhibited longer retention in the tumour. This prolonged retention of PLys(Gln)-100 can be attributed to its higher binding affinity to the tumour cells, which is in line with the apparent *K*_d_ values.

## Discussion

Previous studies revealed potential of aberrant glutamine metabolism as a target of cancer therapy and diagnosis^4, 7–11, 31^, and this aberrant metabolism accompanies overexpression of the corresponding amino acid transporters including ASCT2^6–8^ as we also demonstrated in *in vitro* and *in vivo* (Fig. 2 and Supplementary Fig. S13). While previous research utilized increased cellular uptake of glutamine to visualize accelerated metabolism^10, 11, 31^, this study focused on these overexpressed transporters on tumour cells, and developed the polymer functionalized with glutamine molecules for exerting selective affinity to tumour cells through multivalent effect.

Although exact mechanism of interaction between ASCT2 and glutamine or glutamine-analogues is not fully understood, a previous study has proposed key structures of glutamine-analogues to get a high affinity to ASCT2: α-amine and α-carboxyl groups for charge interaction, γ-amide group for hydrogen binding, and modified side chain for hydrophobic interaction^17^. As most potent ASCT2 inhibitors have α-amine and α-carboxyl groups, these chemical structures appeared to be important for glutamine-ASCT2 interaction. Indeed, PLys(Gln)-100 that has α-amine and α-carboxyl groups at the side chain showed higher affinity both in *in vitro* and *in vivo* compared to PLys(α-Glu)-100 although the difference between these polymers is just the position of amine group at the side chain. In addition to α-amine and α-carboxyl groups, glutamine-like structure should be important for achieving ASCT2 selective interaction because amino acids transporters generally recognize these functional groups. For instance, α-amine and α-carboxyl groups are key components for the substrates of system L transporter 1 (LAT1)^32^, which is overexpressed on a variety of tumour cells^33–35^. Given that LAT1 is reported to be expressed on BxPC3 cells^36^, PLys(Gln)-100 was expected to interact with LAT1 as well as ASCT2 if amino acids transporters recognize just the side chain terminus of the polymers. However, as shown in Fig. 4a, LAT1 inhibitor BCH did not inhibit the PLys(Gln)-100 uptake in BxPC3 cells while ASCT2 inhibitor BzlSer strongly inhibited the uptake. This ASCT2-selective interaction of PLys(Gln)-100 suggests that ASCT2 may recognize not merely α-amine and α-carboxyl groups but whole glutamine-like structure (including γ-amide groups) of the side chain.

On the other hand, given that PLys(Gln)-100 and PLys(α-Glu)-100 has the same valency and density of the binding motifs, moderate interaction between PLys(α-Glu)-100 and ASCT2 might be attributed to the recognition of glutamate-like structure at the side chain by ASCT2. This explanation is consistent with previous reports, where glutamate did not interact with system L and system N glutamine transporters but weakly interacted with ASCT2^22, 23, 27, 37^. In addition, this glutamate-ASCT2 interaction was reported to be pH-dependent; a decrease of pH could enhance the interaction capacity of glutamate with ASCT2^17, 23^. In line with this report, PLys(α-Glu)-100 showed higher uptake in BxPC3 cells at pH 6.5 compared to that at pH 7.4 (Supplementary Fig. S20). However, this increased cellular uptake of PLys(α-Glu)-100 at pH 6.5 was still lower than that of PLys(Gln)-100, indicating that even at the intratumoral pH (~ 6.5) PLys(Gln)-100 should be the preferred structure for achieving high affinity to ASCT2.

As aforementioned, the fine-tuning of DP and chemical structure at the side chain permits the selective interaction of the polymer to dense ASCT2. Given that the overexpression ratio of ASCT2 between BxPC3 and HEK293 cells (Supplementary Fig. S14) is comparable to that in clinical studies^7, 18, 19^, the tumour-selective interaction of the polymer as shown in Fig. 3 strongly indicates the potential *in vivo* activity of our synthesized polymer for tumour-targeting ligand. In our synthetic strategy, the Cy5 fluorescence dye can be easily replaced with the other functional molecules including drugs, diagnostic agents, and functional macromolecules. Hence, the glutamine-based ligand would be potentially useful as a versatile platform for delivering such functional molecules to diseased sites related to aberrant glutamine metabolism. However, systemic application of this polymer still requires optimization. For example, PLys(Gln)-100 would be rapidly eliminated from the body because the molecular weight of the polymer is lower than the threshold of renal clearance^38^; it would have few chances of interacting with tumour tissue after systemic administration. Thus, the design of glutamine-based ligand with suitable architecture for long blood circulation and appropriate binding affinity to the target cell is our future interests.

In summary, we developed the novel glutamine-functionalized polymer with a high affinity to tumour cells overexpressing glutaminolysis-related transporter ASCT2. The glutamine-functionalized polymer exhibited enhanced cellular uptake in the cultured cells by strong interaction with ASCT2. Even in *in vivo* environment, the glutamine-functionalized polymer showed prolonged retention at tumour site, indicating the potent binding affinity to tumour. To the best of our knowledge, this is the first report utilizing glutamine as a functional molecule showing a high affinity to tumour cells, inspired by tumour-associated metabolism. This study offers a fundamental molecular design to target dense transporters associated with tumour-specific aberrant metabolisms.

## Methods

### Materials

*N*-Carboxyanhydride of ε-trifluoroacetyl-L-lysine (NCA-Lys(TFA)) was purchased from Chuo Kasei Co., Ltd. (Osaka, Japan). MeAIB was purchased from Tokyo Chemical Industry Co., Ltd. (Tokyo, Japan). 11-Azido-3,6,9-trioxaundecan-1-amine, Boc-L-glutamic acid 1-benzyl ester (Boc-Glu-OBzl), Boc-L-glutamic acid 5-benzyl ester (Boc-Glu(OBzl)-OH), RPMI 1640 medium, Dulbecco’s modified Eagle’s medium (DMEM), penicillin/streptomycin, BCH, and trypsin/EDTA were purchased from Sigma Aldrich Corporation (St. Louis, MO). Dimethylsulfoxide (DMSO), triethylamine (TEA), *N*-methyl-2-pyrolidone (NMP), BzlSer, Gln, 4-(4,6-dimethoxy-1,3,5-triazin-2-yl)-4-methylmorpholinium chloride (DMT-MM), and D-PBS(-) were purchased from Wako Pure Chemical Industries, Ltd. (Osaka, Japan). DMSO was purified by distillation under reduced pressure before use. Dibenzocyclooctyne-Cy5 (Cy5-DBCO) was purchased from Click Chemistry Tools (Scottsdale, AZ). Fetal bovine serum (FBS), and Hoechst 33342 were purchased from Thermo Fisher Scientific, Inc. (Waltham, MA). Bovine serum albumin (BSA) was purchased from Nacalai Tesque, Inc. (Kyoto, Japan).

### Cell lines and animals

BxPC3 cells, HEK293 cells, and HepG2 cells were purchased from ATCC (Manassas, VA). BxPC3 cells were cultured under a humidified atmosphere containing 5% CO_2_ at 37 °C in RPMI 1640 supplemented with 10% FBS and 1% penicillin/streptomycin. HEK293 cells and HepG2 cells were cultured under a humidified atmosphere containing 5% CO_2_ at 37 °C in DMEM supplemented with 10% FBS and 1% penicillin/streptomycin. BALB/c nu/nu mice (female, 4 weeks old) were purchased from Charles River Laboratories Japan, Inc. (Yokohama, Japan). All animal experiments were approved by the Animal Care and Use Committee of Tokyo Institute of Technology, and performed in accordance with the Guidelines for the Care and Use of Laboratory Animals as stated by Tokyo Institute of Technology.

### Immunohistochemistry

To establish the BxPC3 xenograft model, the cells (5.0 × 10^6^ cells) were subcutaneously inoculated to BALB/c nu/nu mice. When tumour volume reached approximately 150 mm^3^, the mice were perfused using saline and fixed using 4% paraformaldehyde. Tissues were excised and frozen in OCT compound (Sakura Finetek Japan Co., Ltd., Tokyo, Japan). The frozen samples were sectioned at 4-μm thickness and washed with PBS. After 1 h blocking treatment using 5% skim milk in PBS, the sections were incubated with rabbit anti-human/murine ASCT2 polyclonal antibody (1:100 dilution in PBS containing 5% skim milk, LSBio, Seattle, WA) overnight at 4 °C. Then, the sections were washed with PBS three times, incubated with Alexa Fluor 568-conjugated goat anti-rabbit IgG (1:800 dilution in PBS containing 5% skim milk, Thermo Fischer Scientific) at ambient temperature for 45 min. After washing with PBS three times, the sections were incubated with Hoechst 33342 (1:1000 dilution in PBS) for staining the nuclei, and mounted in Vectashield mounting media (Vector Laboratories, Burlingame, CA). The obtained tissue sections were observed using a fluorescence microscope (BZ-X710, Keyence, Osaka, Japan).

### Flow cytometric analysis of ASCT2 expression

BxPC3 cells and HEK293 cells (2.0 × 10^5^ cells) were transferred into 1.5 mL tube and washed with assay buffer (PBS containing 1% BSA). Then, the cells were resuspended in 100 μL of rabbit anti-human ASCT2 polyclonal antibody solution (1:50 dilution in the assay buffer, Santa Cruz Biotechnology, Dallas, TX) or isotype control rabbit IgG solution (1:250 dilution in the assay buffer, abcam, Cambridge, MA) and incubated for 1.5 h on ice. The cells were washed twice with 500 μL of the assay buffer and resuspended in 100 μL of DyLight 650-conjugated anti-rabbit IgG solution (1:500 dilution in the assay buffer, abcam). After 45 min incubation on ice in the dark, the cells were washed twice with 500 μL of the assay buffer and resuspended in the 500 μL of the assay buffer, followed by analysis using flow cytometer (Guava easyCyte 6-2 L, Merck Millipore, Billerica, MA) (ex/em = 642 nm/661 nm).

### Synthesis of azide-functionalized poly(L-lysine) (azide-PLys)

A series of azide-PLys-n (n is the polymerization degree of Lys unit) were synthesized by ring-opening polymerization of NCA-Lys(TFA) and subsequent deprotection of trifluoroacetyl group (Supplementary Fig. S1). Briefly, for the synthesis of azide-PLys-100, the NCA-Lys(TFA) (2.16 g, 8.1 mmol) was polymerized in distilled DMSO (10 mL) initiated by 11-azido-3,6,9-trioxaundecan-1-amine (16 μl, 0.081 mmol) at room temperature for 3 days under argon atmosphere. The polymer was purified by dialysis against MeOH (MWCO: 1,000). The dialyzed solution in MeOH was then evaporated and dried *in vacuo* to obtain azide-PLys(TFA)-100 (1.73 g, yield = 92%). The polydispersity (*M*_w_/*M*_n_) of the obtained polymer was determined to be 1.2 by gel permeation chromatography [column: TSK-gel superAW3000, superAW4000, and superAWL-guard column (Tosoh Corporation, Yamaguchi, Japan); eluent: NMP containing 50 mM LiBr; flow rate: 0.3 ml/min; detector: refractive index (RI); temperature: 40 °C]. Then, the obtained azide-PLys(TFA)-100 (1.0 g) was dissolved in 5 N NaOH aq./MeOH (1:4 (v/v), 20 mL) and stirred at room temperature for 8 h to deprotect trifluoroacetyl group. The reaction mixture was purified by dialysis against 0.01 N HCl aqueous solution and subsequent deionized water (MWCO: 1,000), followed by lyophilisation to afford azide-PLys-100 (0.545 g, yield = 74%). The degree of polymerization was calculated to be 103 from the proton ratio of the ethylene groups of initiator (δ = 3.4–3.8 ppm) to the butylene groups of Lys (δ = 1.3–1.9 and 2.9 ppm) in the ^1^H NMR (AVANCE III 400, Bruker, Billerica, MA) spectrum (solvent: D_2_O). ^1^H NMR (400 MHz, D_2_O): δ(ppm) = 1.3–1.9 (618 H, -C***H***_2_C***H***_2_C***H***_2_CH_2_NH_3_), 2.9 (206 H, -CH_2_CH_2_CH_2_C***H***_2_NH_3_), 3.4–3.8 (16 H, -C***H***_2_C***H***_2_O- of initiator), 4.3 (103 H, -COC***H***NH-). Azide-PLys-30 and azide-PLys-50 were synthesized in the same procedure. The polydispersity index (*M*_w_/*M*_n_) of both azide-PLys(TFA)-30 and azide-PLys(TFA)-50 was determined to be 1.1. The degree of polymerization of azide-PLys-30 and azide-PLys-50 was calculated to be 29 and 51, respectively.

### Synthesis of glutamine-modified azide-PLys (azide-PLys(Gln)) and α-glutamate-modified azide-PLys (azide-PLys(α-Glu))

Azide-PLys(Gln) and azide-PLys(α-Glu) were synthesized by condensation reaction and deprotection reaction (Supplementary Fig. S2, S3). For the synthesis of azide-PLys(Gln), Boc-Glu-OBzl (121 mg, 0.36 mmol) and DMT-MM (249 mg, 0.90 mmol) were added to a solution of azide-PLys-100 (30 mg, 0.0017 mmol) in a mixture of DMSO (5 mL) and TEA (0.5 mL). The reaction mixture was stirred overnight at room temperature, and then purified by dialysis against MeOH (MWCO: 6–8,000). After dialysis, the residue was evaporated and dried *in vacuo* to obtain azide-PLys(Boc-Glu-OBzl)-100 in powder form (83 mg, yield > 99%). Quantitative installation of Boc-Glu-OBzl was confirmed by the proton ratio of phenyl group of Boc-Glu-OBzl (δ = 7.3 ppm) and methylene group of Lys (δ = 3.1 ppm) in the ^1^H NMR spectrum (solvent: MeOD). To deprotect Bzl group, the obtained polymer was dissolved in a mixture of 0.5 N NaOH aq. (3 mL) and MeOH (3 mL). After stirring at room temperature for 18 h, the reaction mixture was purified by dialysis against deionized water (MWCO: 6–8,000), followed by lyophilisation. Complete deprotection of Bzl group was confirmed by ^1^H NMR spectrum (solvent: D_2_O). To deprotect the Boc group, the polymer was dissolved in a mixture of 3 N HCl (3 mL) and AcOH (3 mL), and stirred at room temperature for 18 h. The reaction mixture was purified by dialysis against deionized water (MWCO: 6–8,000) and freeze-dried to afford azide-PLys(Gln)-100 (46 mg, yield = 85%). ^1^H NMR (400 MHz, D_2_O): δ(ppm) = 1.3–1.9 (618 H, -C***H***_2_C***H***_2_C***H***_2_CH_2_NHCO-), 2.1–2.4 (412 H, -NHCOC***H***_2_C***H***_2_CH-), 3.1 (206 H, -CH_2_CH_2_CH_2_C***H***_2_NH_3_), 3.4–3.7 (16 H, -C***H***_2_C***H***_2_O- of initiator), 4.3 (103 H, -COC***H***NH-), 3.8 (103 H, -NHCOCH_2_CH_2_C***H***-). Azide-PLys(Gln)-50 and azide-PLys(Gln)-30 were synthesized in the same procedure. For the synthesis of azide-PLys(α-Glu), Boc-Glu(OBzl)-OH was used instead of Boc-Glu-OBzl.

### Cy5 conjugation to azide-PLys(Gln) and azide-PLys(α-Glu)

Dibenzocyclooctyne-Cy5 dye (Cy5-DBCO) was conjugated to azide-PLys(Gln) and azide-PLys(α-Glu) by copper-free click chemistry to afford PLys(Gln) and PLys(α-Glu) as shown in Supplementary Fig. S4, S5. For the synthesis of PLys(Gln)-100, azide-PLys(Gln)-100 (46 mg, 0.0015 mmol) and Cy5-DBCO (0.5 mg, 0.00050 mmol) was dissolved in 10 mM NaHCO_3_ aqueous solution (2 mL, pH 7.4) and stirred overnight at room temperature. The reaction mixture was purified by dialysis against deionized water (MWCO: 6–8,000). After freeze-drying, the crude product was dissolved in 1 M NaCl aqueous solution and further purified by PD-10 column (Sephadex G-25, GE Healthcare Ltd., UK), followed by dialysis against deionized water (MWCO: 6–8,000) and lyophilisation. The PLys(Gln)-100 was obtained as a blue powder (32 mg, yield = 70%). PLys(Gln)-n (n = 50, 30) or PLys(α-Glu)-n (n = 100, 50, 30) were synthesized in the same procedure. The final products were characterized by size exclusion chromatography [column: Superdex 200 increase 10/300GL (GE Healthcare Ltd); eluent: 10 mM phosphate buffer (pH 7.4) containing 150 mM NaCl; flow rate: 0.6 ml/min; temperature: room temperature; detector: fluorescence (ex/em = 620 nm/670 nm)].

### Cellular uptake analysis

BxPC3 cells, HEK293 cells, and HepG2 cells (5.0 × 10^4^ cells/well) were seeded into 24-well plates and incubated at 37 °C for 24 h. The cells were washed with PBS, and incubated in 500 μl of assay buffer (PBS containing 10% FBS) with 1 μM of PLys(Gln)-n or PLys(α-Glu)-n. After 3 h incubation at 37 °C, the cells were washed twice with PBS, treated with 150 μL of trypsin, and suspended with 300 μl of the assay buffer. Cy5 fluorescence intensities of the cells were measured using the flow cytometer (ex/em = 642 nm/664 nm).

### Time-dependent cellular uptake analysis

The procedure of time-dependent cellular uptake study was same in cellular uptake study described above except for incubation time with the polymers. Incubation time with the polymers was changed to 1, 2, 4, or 8 h.

### Cellular uptake analysis with inhibitors

Cellular uptake of the polymers was evaluated in the presence of specific inhibitors (BzlSer, ASCT2 inhibitor; MeAIB, system A inhibitor; BCH, system L inhibitor; Gln, system N inhibitor) in a concentration ranging from 2.5 mM to 10 mM. The procedure was same as the cellular uptake study except for the addition of inhibitors to the polymer solution. The data are expressed as a percentage of fluorescence intensity obtained from the cells treated without any inhibitor.

### Confocal laser scanning microscopic observation

To visualize the subcellular distribution of the polymers, BxPC3 cells treated with each polymer at 37 °C or 4 °C were observed using a confocal laser scanning microscope (LSM710, Carl Zeiss, Oberkochen, Germany). BxPC3 cells (5 × 10^4^ cells/dish) were seeded into 35 mm glass-based dish (Asahi Glass Co., Ltd., Tokyo, Japan) and incubated at 37 °C for 24 h. For the treatment at 37 °C, the cells were washed with PBS, and incubated in 1 mL of PBS with 10% FBS and 1 μM of the polymer at 37 °C for 2.5 h. To stain late endosome/lysosome and nuclei, LysoTracker Red (Thermo Fischer Scientific) and Hoechst 33342 were added to the solution. After additional 30 min incubation at 37 °C, the cells were washed twice with PBS, and observed in fresh medium using LSM710 equipped with an incubator (37 °C, 5% CO_2_ in humidified atmosphere). For the treatment at 4 °C, the cells were cooled at 4 °C for 10 min and washed with cold PBS, followed by incubation in 1 mL of PBS with 10% FBS and 1 μM of the polymers at 4 °C for 2.5 h. Then, LysoTracker Red and Hoechst 33342 were added to the solution, and the cells were incubated at 4 °C for another 30 min. The cells were washed twice with cold PBS, fixed with 4% paraformaldehyde in PBS, and observed in fresh PBS using LSM710. Cy5, LysoTracker Red, and Hoechst 33342 were excited using laser light at 633 nm, 561 nm, and 405 nm, respectively.

### Cellular uptake at 4 °C

BxPC3 cells (5.0 × 10^4^ cells/well) were seeded into 24-well plates and incubated at 37 °C for 24 h. The cells were cooled at 4 °C for 10 min and washed with cold PBS, followed by the incubation in 500 µl of the assay buffer (10% FBS in PBS) containing 1 μM of the polymers. After 3 h incubation at 4 °C, the cells were washed twice with cold PBS, detached with 150 μL of trypsin, and suspended with 300 μL of the assay buffer. The cells were analysed using flow cytometer, and the obtained Cy5 fluorescence intensities were expressed as a percentage of the fluorescence intensity obtained from cells treated at 37 °C.

### Cell-based competitive inhibition

BxPC3 cells (5.0 × 10^4^ cells/well) were seeded into 24-well plates and incubated at 37 °C for 24 h. For the inhibition of PLys(Gln)-100, the cells were cooled at 4 °C for 10 min, washed with cold PBS, followed by incubation at 4 °C for 2 h in 500 μL of the assay buffer (10% FBS in PBS) containing 1 μM of PLys(Gln)-100 and BzlSer in a concentration ranging from 30 mM to 0.04 mM. After the incubation, the cells were washed with cold PBS, treated with 150 μL of trypsin, and suspended with 300 μL of the assay buffer. The cells were analysed using flow cytometer, and half maximal inhibitory concentrations (IC_50_) of BzlSer were calculated. Apparent dissociation constant (*K*_d_) of the polymers were estimated using the obtained IC_50_ values, following Cheng-Prusoff equation (1)^30, 39^:

1 $${K}_{d}={K}_{i}\times [L]/(I{C}_{50}-{K}_{i})$$

where *K*_i_ is the apparent inhibition constant of BzlSer for ASCT2 (0.9 mM), and [L] is the polymer concentration in this assay. For the inhibition of PLys(Gln)-50 and PLys(α-Glu)-100, concentration of both polymers was changed to 3 μM.

### *In vivo* tumour retention

To establish the BxPC3 xenograft model, the cells (5.0 × 10^6^ cells) were subcutaneously inoculated to BALB/c nu/nu mice (female, 4 weeks old). When average tumour volume reached approximately 150 mm^3^, 20 μl of PLys(Gln)-100, PLys(Gln)-50, or PLys(α-Glu)-100 solution (40 μM in PBS) was intratumorally injected (n = 3). At 0.25, 1, 4, and 8 h after injection, the mice were imaged by *in vivo* imaging system (IVIS, Perkin Elmer, Waltham, MA) using 640 nm/680 nm ex/em filter. Average radiant efficiency of the tumour region was quantified using Living Image software.

### Statistical analysis

Statistical analysis was performed using Student’s *t*-test for the comparison of the mean between two groups, and ANOVA with Tukey’s multiple comparison test for the comparison of the mean among more than three groups. The *p* values less than 0.05 were considered as statistically significant.

## Electronic supplementary material

Supplementary Information
