# Supplementary material for: Engineering Tumour Cell-Binding Synthetic Polymers with Sensing Dense Transporters Associated with Aberrant Glutamine Metabolism
Source: Sci Rep. 2017 Jul 20;7:6077. doi: 10.1038/s41598-017-06438-y (PMC5519697; doi:10.1038/s41598-017-06438-y)
Supplement: Supplementary file 1 — Supplementary Information [file 41598_2017_6438_MOESM1_ESM.pdf]

Supplementary Information

Engineering Tumour Cell-Binding Synthetic Polymers with Sensing Dense Transporters Associated with Aberrant Glutamine Metabolism

Naoki Yamada, Yuto Honda, Hiroyasu Takemoto, Takahiro Nomoto, Makoto Matsui, Keishiro Tomoda, Masamitsu Konno, Hideshi Ishii, Masaki Mori, and Nobuhiro Nishiyama*

Supplementary Figures

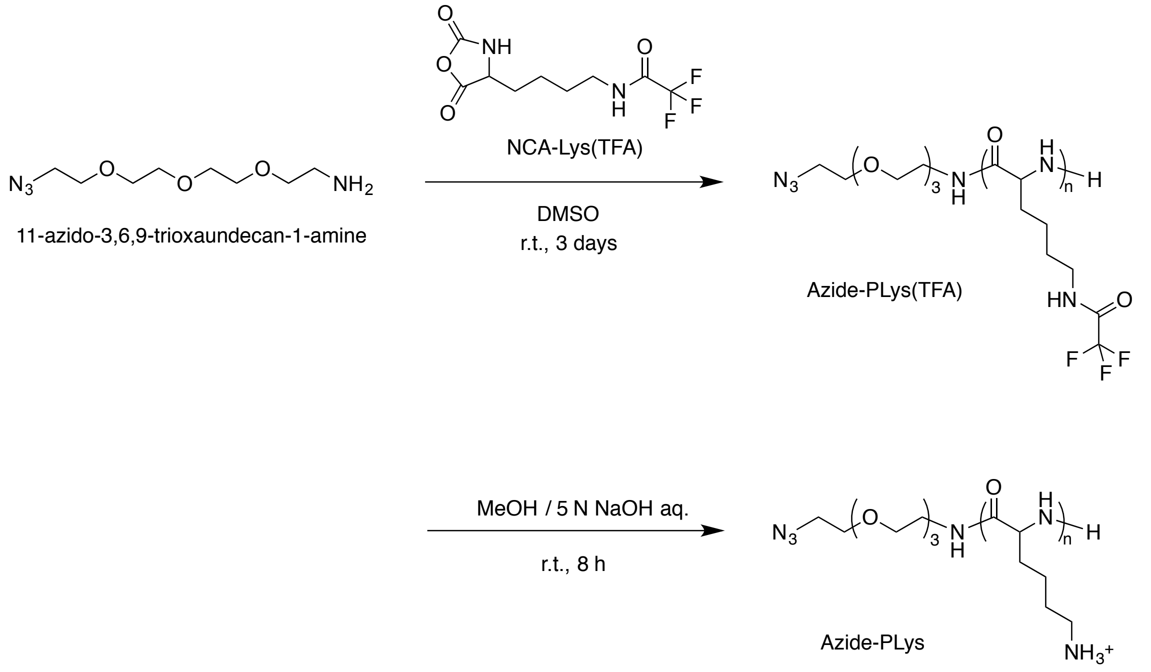

Supplementary Figure S1. Synthetic procedure for azide-PLys.

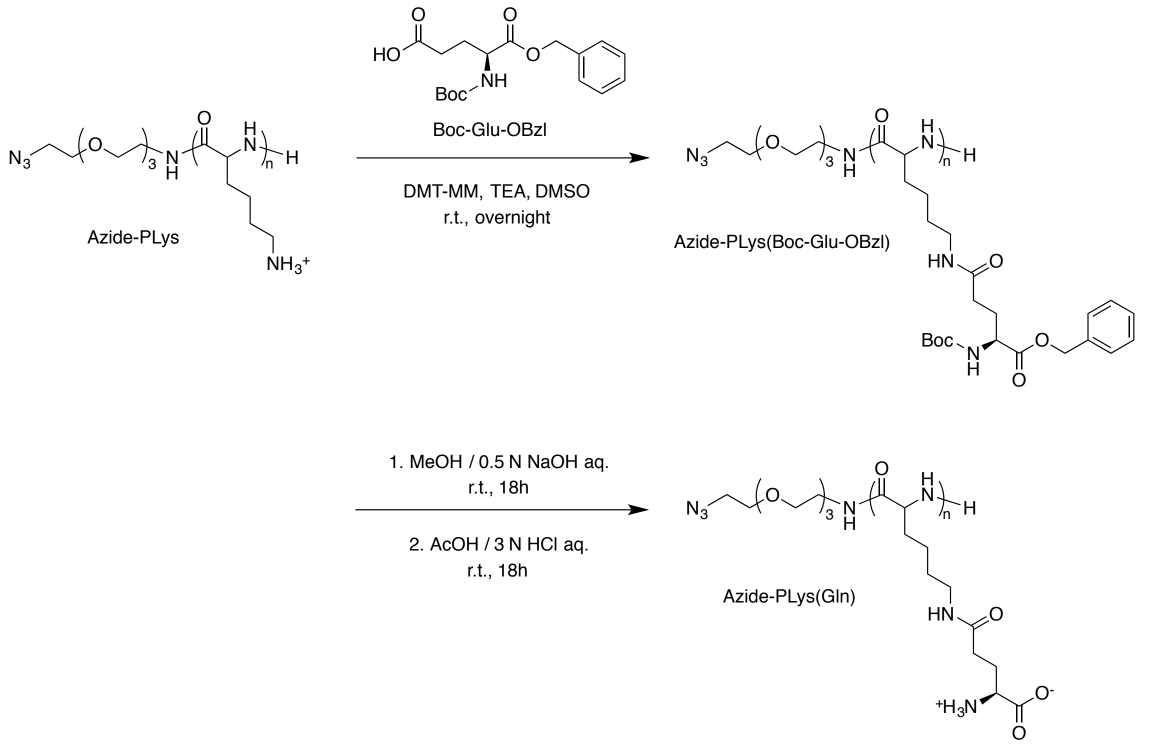

Supplementary Figure S2. Synthetic procedure for azide-PLys(Gln).

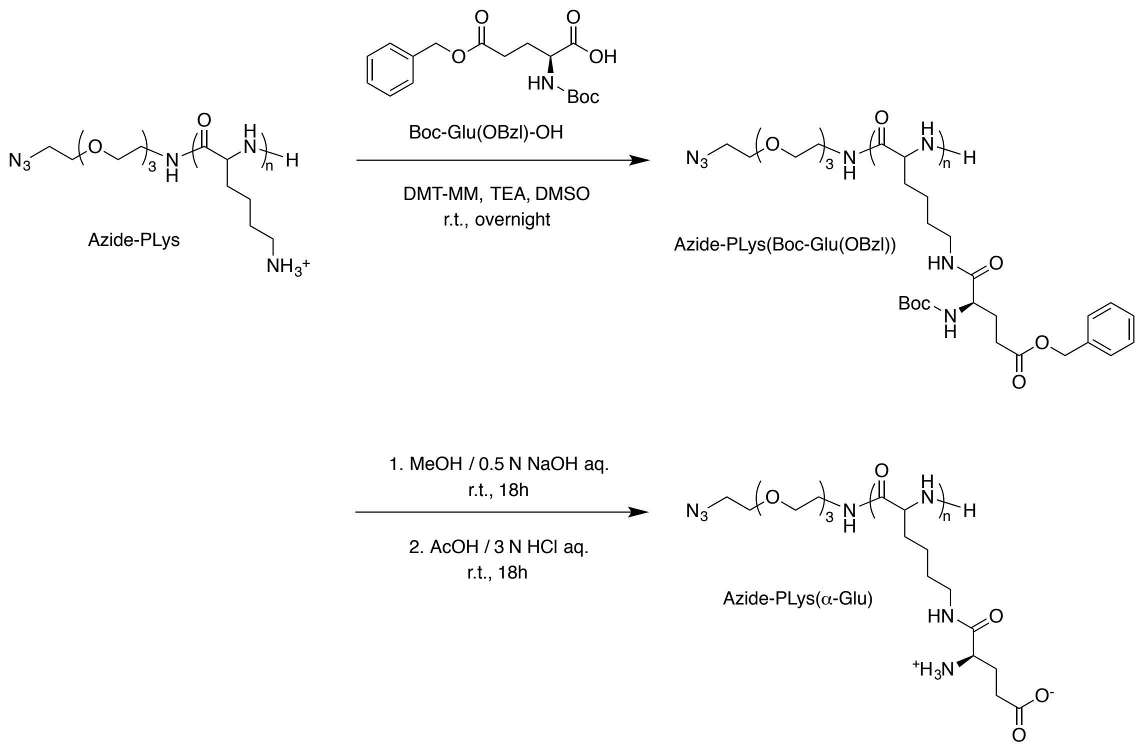

Supplementary Figure S3. Synthetic procedure for azide-PLys(α-Glu).

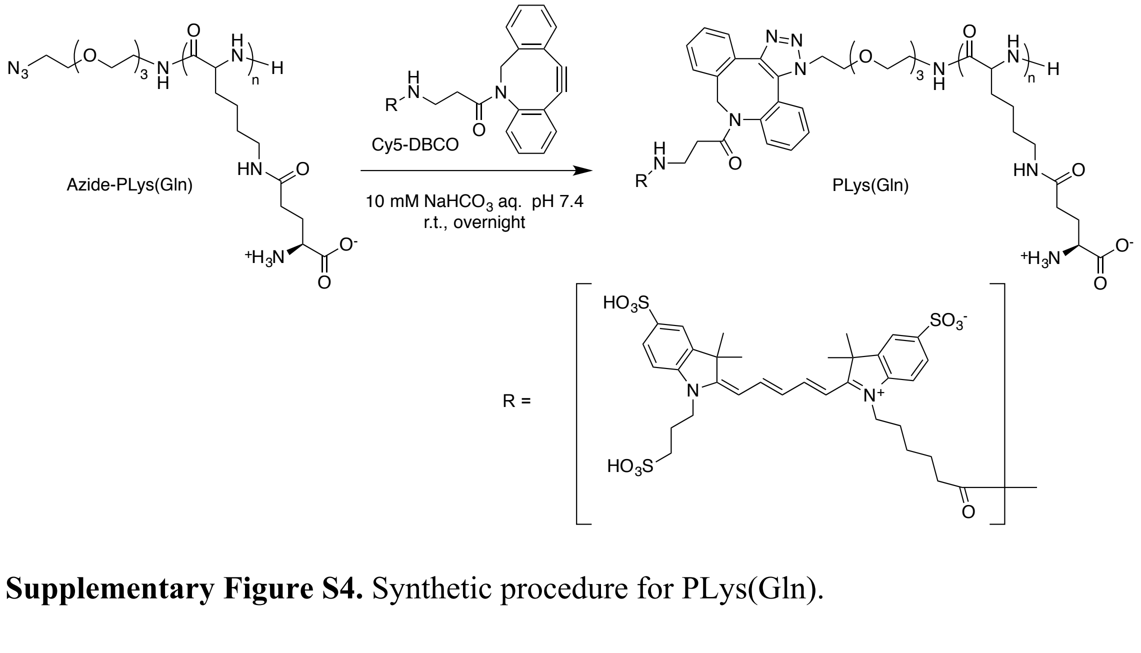

Supplementary Figure S4. Synthetic procedure for PLys(Gln).

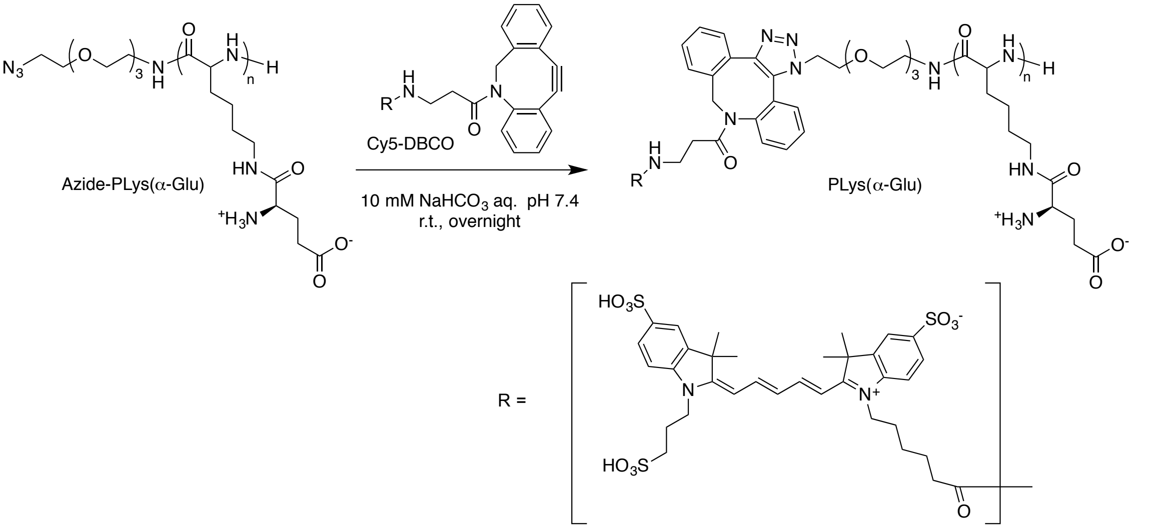

Supplementary Figure S5. Synthetic procedure for PLys(α-Glu).

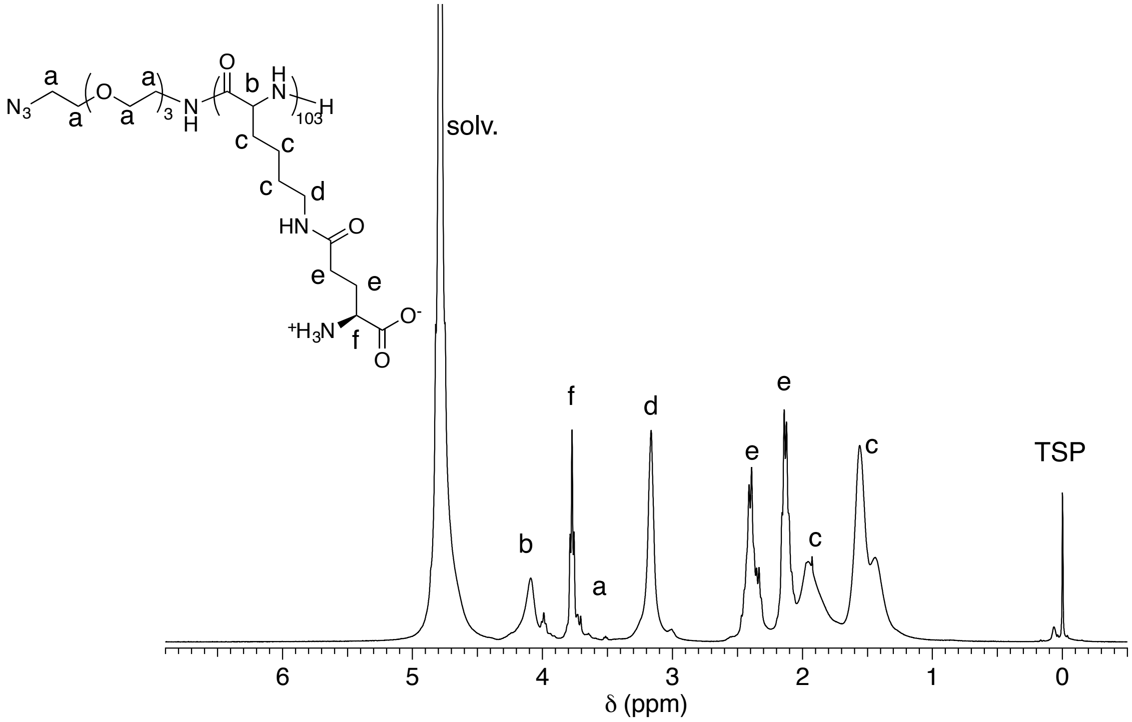

Supplementary Figure S6. ^{1}H NMR spectrum of azide-PLys(Gln)-100 (D_2O).

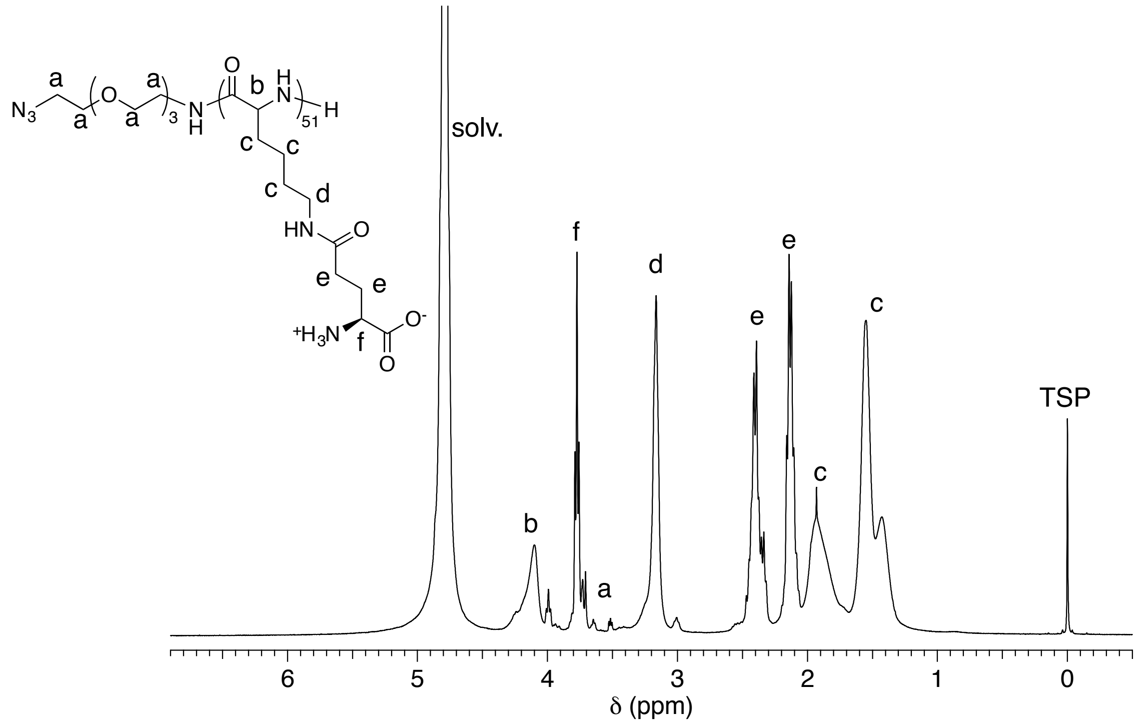

Supplementary Figure S7. ^{1}H NMR spectrum of azide-PLys(Gln)-50 (D_2O).

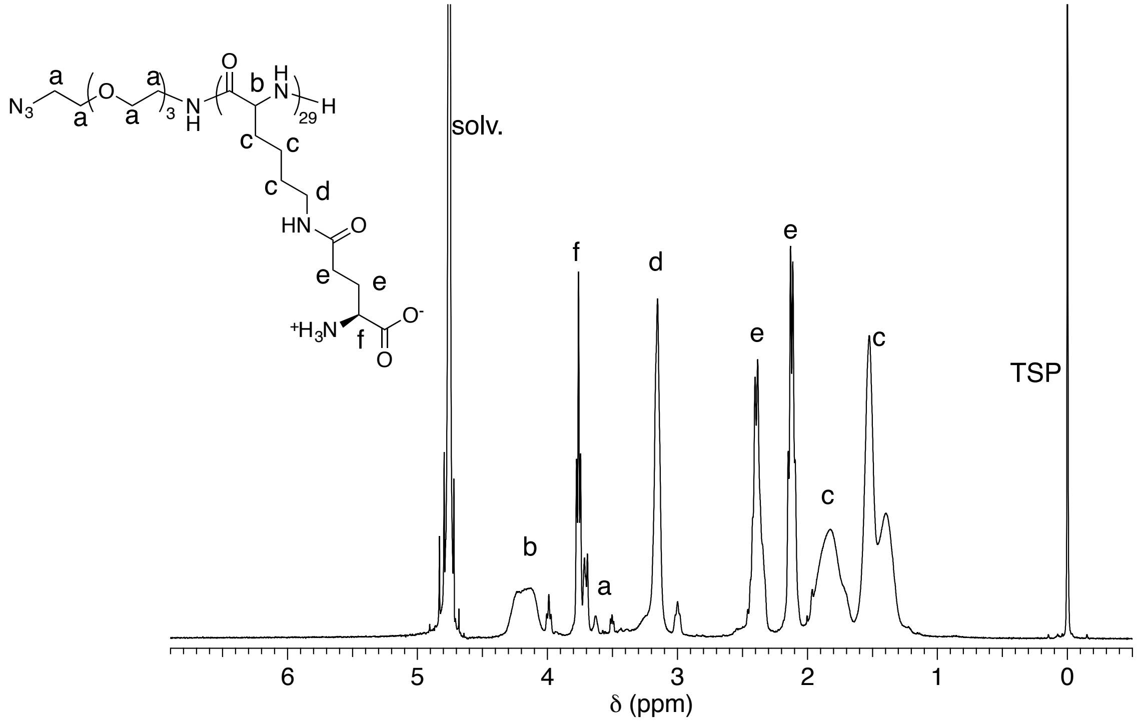

Supplementary Figure S8. ^{1}H NMR spectrum of azide-PLys(Gln)-30 (D_2O).

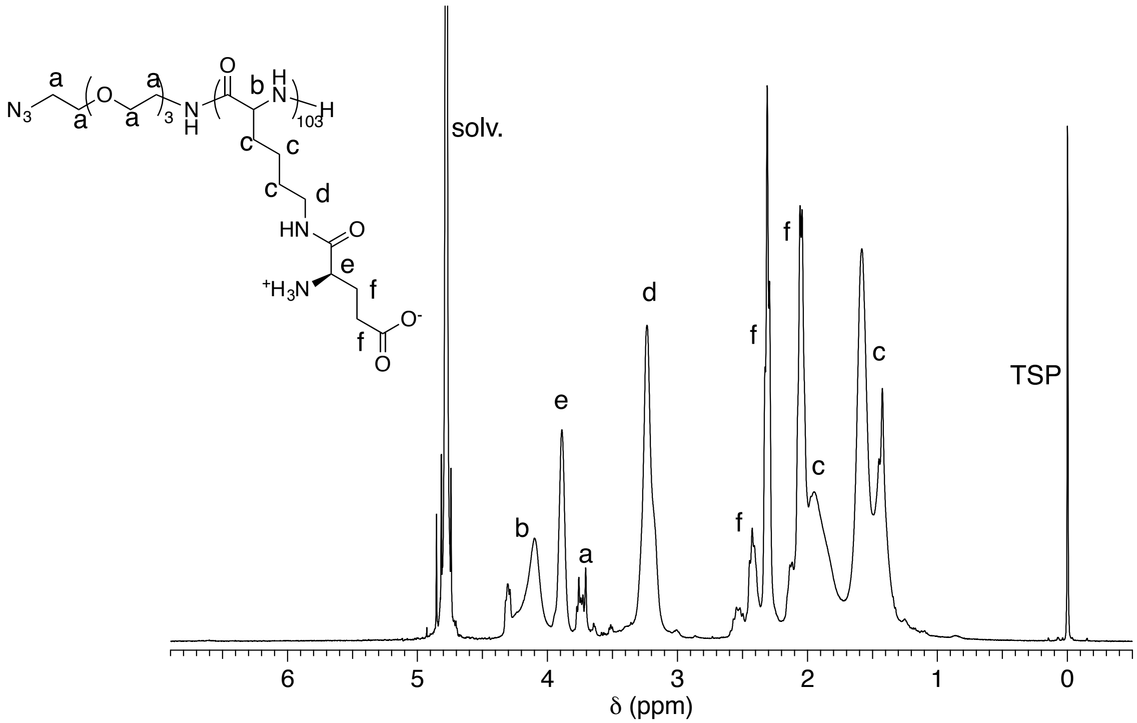

Supplementary Figure S9. ^{1}H NMR spectrum of azide-PLys(α-Glu)-100 (D_2O).

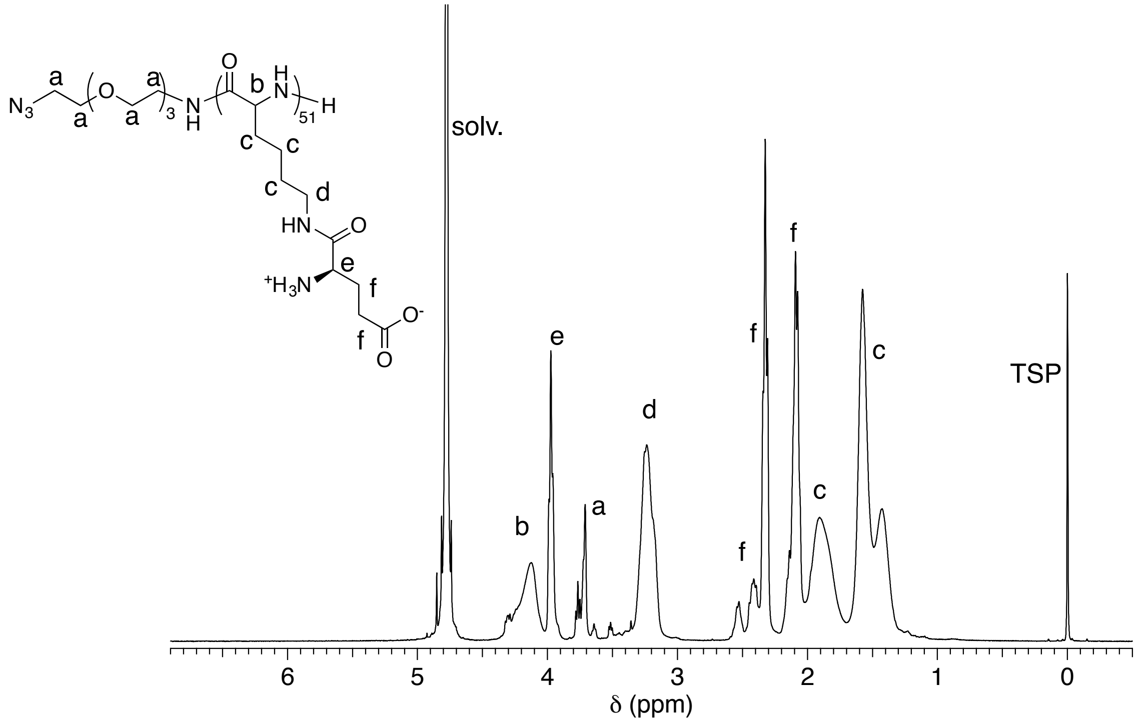

Supplementary Figure S10. ^{1}H NMR spectrum of azide-PLys(α-Glu)-50 (D_2O).

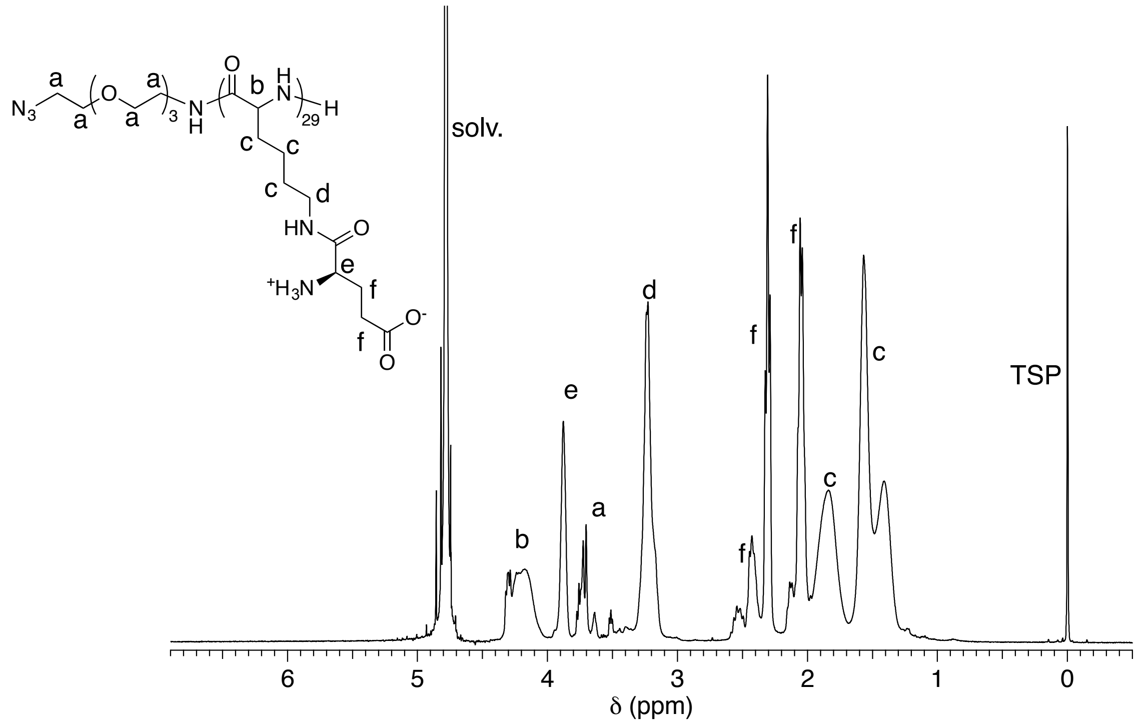

Supplementary Figure S11. ^{1}H NMR spectrum of azide-PLys(α-Glu)-30 (D_2O).

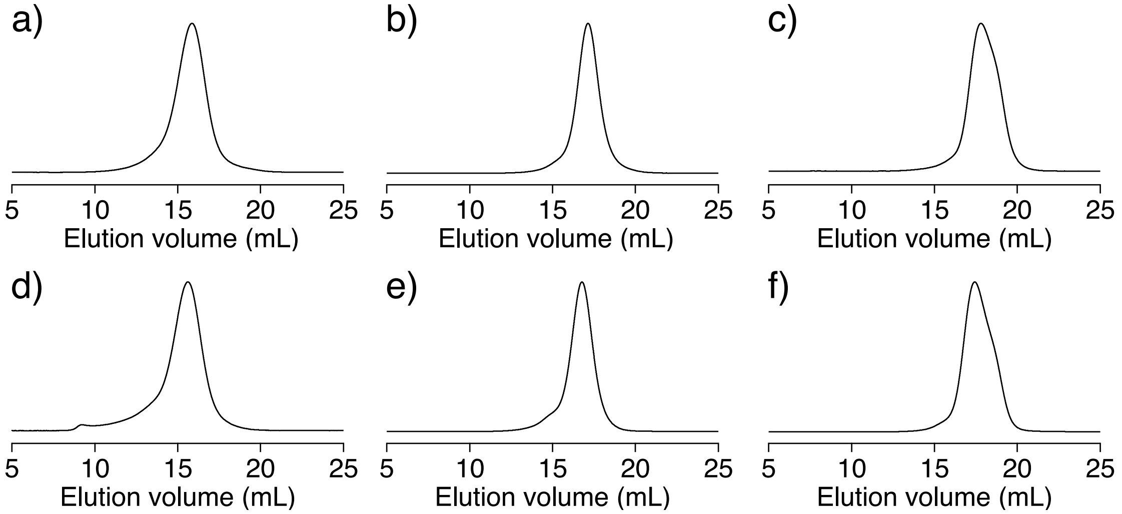

Supplementary Figure S12. Size exclusion chromatography of a series of PLys(Gln) and PLys(α-Glu). a) PLys(Gln)-100, b) PLys(Gln)-50, c) PLys(Gln)-30, d) PLys(α-Glu)-100, e) PLys(α-Glu)-50, f) PLys(α-Glu)-30. [column, Superdex 200 increase 10/300GL; eluent, 10 mM phosphate buffer (pH 7.4) containing 150 mM NaCl; flow rate, 0.6 ml/min; temperature, r.t.; detector, fluorescence (ex/em = 620 nm/670 nm)]

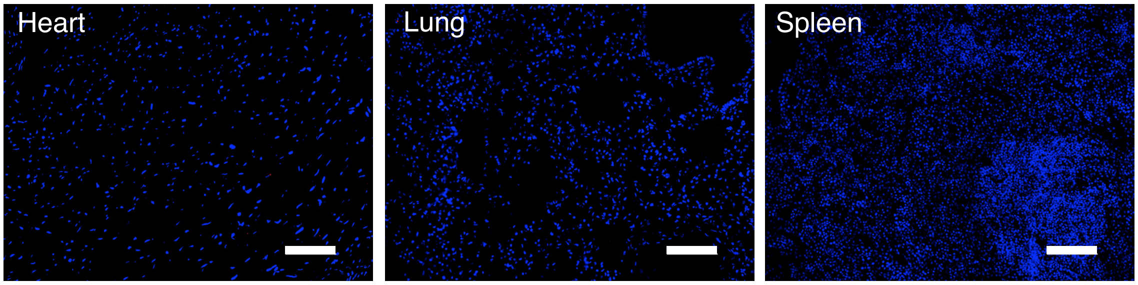

Supplementary Figure S13. Immunohistochemical analysis of tissues from mice bearing subcutaneous BxPC3 tumours. Red, anti-human/murine ASCT2 antibody; blue, nuclei. Scale bar, 100 μm.

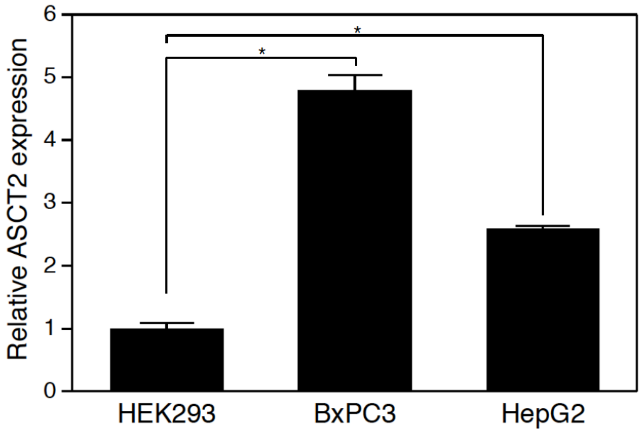

Supplementary Figure S14. Relative expression level of ASCT2 quantified by ELISA. Data are mean ± S.D. (n=3). $p^* < 0.001$ (one-way ANOVA with Tukey's multiple comparison test).

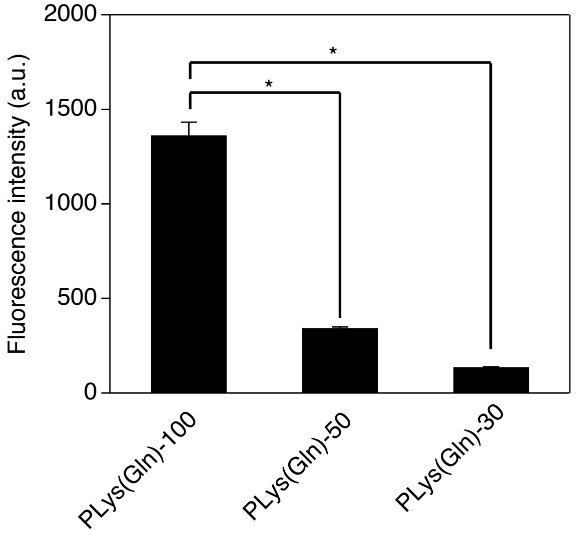

Supplementary Figure S15. Cellular uptake analysis of polymers in HepG2 cells. The cells were incubated with the polymers at 37˚C for 3 h. Data are mean ± S.D. (n=3). $p^* < 0.001$ (one-way ANOVA with Tukey's multiple comparison test)

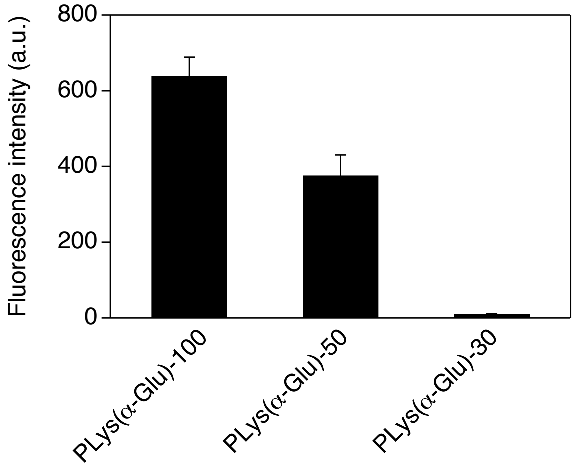

Supplementary Figure S16. Cellular uptake analysis of a series of PLys(α-Glu)-n in BxPC3 cells. The cells were incubated with the polymers at 37 ˚C for 3h. Data are mean ± S.D. (n=3).

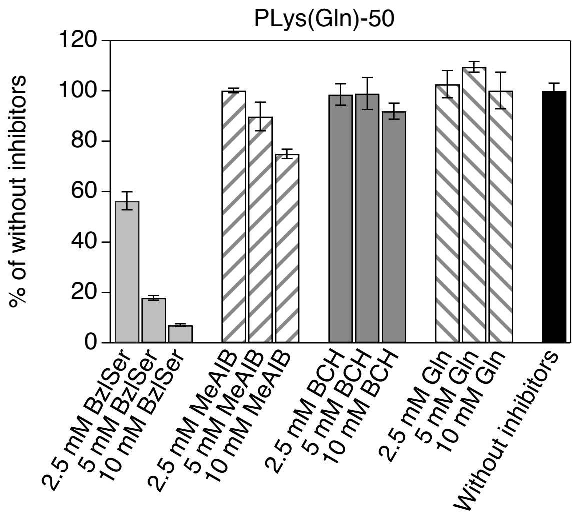

Supplementary Figure S17. Cellular uptake analysis of PLys(Gln)-50 in BxPC3 cells with inhibitors (BzlSer, ASCT2 inhibitor; MeAIB, system A inhibitor; BCH, system L inhibitor; Gln, system N inhibitor). Data are mean ± S.D. (n=3).

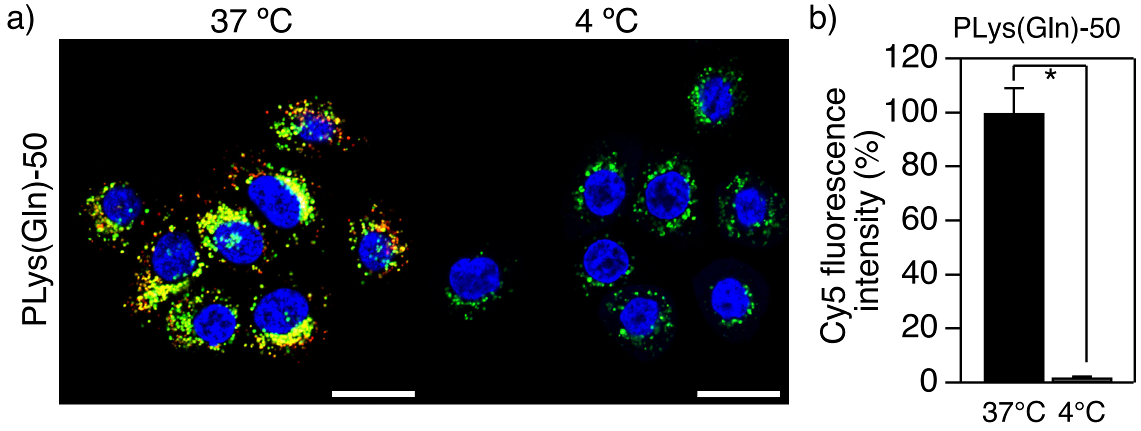

Supplementary Figure S18. a) Confocal laser scanning microscopic images of BxPC3 cells treated with PLys(Gln)-50 at 37 ˚C (left) and 4 ˚C (right). Red, Cy5-labeld polymers; green, late endosome/lysosome; blue, nucleus. Scale bar, 20 mm. b) Cy5 fluorescence intensities of BxPC3 cells after 3 h treatment with PLys(Gln)-50 at 37 °C and 4 °C. Data are mean ± S.D. (n=3). $p^* < 0.001$ (Student's *t*-test).

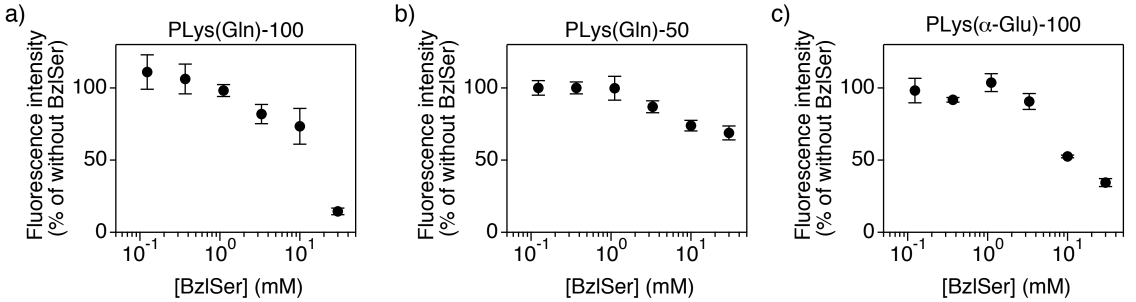

Supplementary Figure S19. Cell-based competitive inhibition curve of PLys(Gln)-100 (a), PLys(Gln)-50 (b), and PLys(α-Glu)-100 (c). Data are mean ± S.D. (n=3).

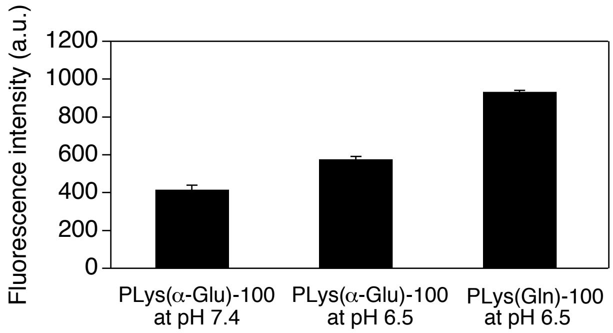

Supplementary Figure S20. Cellular uptake analysis of PLys(Gln)-100 and PLys(α-Glu)-100 in BxPC3 cells at pH 6.5. Data are mean ± S.D. (n=3).

Supplementary Methods

ELISA

The relative expression level of ASCT2 in cultured cells was quantified using ELISA Starter Accessory Kit (Bethyl Laboratories, Inc., Montgomery, TX) according to the manufacture's procedure. Briefly, the cells (5.0×10^6 cells) were transferred to 1.5 mL tubes and washed three times with cold PBS. The cells were then lysed by sonication in 500 μL of lysis buffer (TBS containing 1 mM EDTA, 0.1% NP-40 (Sigma Aldrich Corporation), and protease inhibitor cocktail (Roche, Mannheim, Germany), pH 7.5). The resulting solutions were centrifuged (4˚C, 10,000 g, 15 min) to collect the supernatants. Protein concentration of the collected supernatants was quantified using BCA assay (Thermo Fischer Scientific). To perform the ELISA, microtiter plates were coated with 50 μg of the protein lysates by incubating overnight at 4 ˚C. The plates were washed three times with washing buffer (TBS with 0.05% Tween 20, pH 8.0) and treated with 1% BSA in TBS at ambient temperature for 30 min. After washing three times with washing buffer, the plates were incubated with 100 μL of rabbit anti-human ASCT2 polyclonal antibody solution (1:300 dilution in TBS containing 1% BSA and 0.05% Tween 20, Santa Cruz Biotechnology) at ambient temperature for 1.5 h. The plates were washed three times with washing buffer and incubated with 100 μL of horseradish peroxidase (HRP)-conjugated goat anti-rabbit IgG antibody (1:800 dilution in TBS containing 1% BSA and 0.05% Tween 20, Thermo Fischer Scientific) at ambient temperature for 1 h. After washing five times with washing buffer, 100 μL of 3,3',5,5'-tetramethylbenzidine (TMB) substrate solution was added to each wells for the reaction with HRP. The reaction was stopped by the

addition of 100 μL of 0.18 M H_2SO_4, and the absorbance at 450 nm was measured.

Cellular uptake analysis at pH 6.5

To investigate the effect of pH on the cellular uptake behavior of PLys(α-Glu)-100 and PLys(Gln)-100 in BxPC3 cells, the pH of the assay buffer (PBS containing 10% FBS) was adjusted to 6.5 using HCl aqueous solution. The procedure was same in cellular uptake study described above except for the pH of the assay buffer.
